# “It’s two separate systems that … keep you under a thumb”: dual debt in the child support and criminal legal systems

**DOI:** 10.1017/lsr.2024.47

**Published:** 2024-11-14

**Authors:** Hannah Schwendeman, Veronica L. Horowitz, Frank Edwards, Robert Stewart, Ryan Larson, Christopher Uggen

**Affiliations:** 1 Department of Sociology, University of Minnesota – Twin Cities, Minneapolis, MN, USA; 2 Department of Sociology, SUNY-University at Buffalo, Buffalo, NY, USA; 3 Rutgers University – Newark, Newark, NJ, USA; 4 Department of Criminology and Criminal Justice, The University of Maryland, College Park, College Park, MD, USA; 5 Department of Criminal Justice and Forensic Science, Hamline University, Saint Paul, MN, USA

**Keywords:** Child support, criminal legal debt, legal cynicism, legal estrangement, system avoidance, debt, poverty, punishment, welfare

## Abstract

People simultaneously entangled in multiple state systems are often subject to contradictory legal mandates that can foster distrust and incentivize system avoidance. This study focuses on those indebted to both the child support system and the criminal legal system, a situation we describe as *dual debt*. We ask whether and how the imposition of legal debts with punitive surveillance and collections mechanisms fosters alienation in the form of legal cynicism and estrangement, which we refer to jointly as legal anomie. Drawing from interview data in Minnesota, we find that legal anomie and system avoidance are mutually reinforcing processes, as debts in these systems triggered consequences that pushed people out of the formal labor market and heightened their distrust of legal institutions. The case of dual debt demonstrates how alienating and contradictory policy systems can foster both legal anomie and system avoidance, particularly in the context of economic and social precarity.

## Introduction

Perceptions of the law influence how individuals engage or avoid the legal system. Individual distrust of and disengagement from the law is a significant area of sociolegal scholarship, broadly theorized as legal cynicism (Sampson and Bartusch 1998) and legal estrangement (Bell 2017). It is well documented that contact with the law can increase negative perceptions of the law and its actors, particularly for race-class subjugated communities that are heavily policed yet under-protected from violence (Kramer and Remster 2022; Soss and Weaver 2017). Importantly, experiences with the law can also impact how individuals engage with other key social institutions. As Brayne (2014) has shown, people with criminal legal contact often disconnect from hospitals, schools, banks, and the formal labor market to avoid further surveillance. Although both distrust of the law and strategic disengagement are well-established within the literature, we know little about how these perceptions and behaviors may operate together. More specifically, sociolegal scholars have identified a broad pattern of both legal cynicism and system avoidance among people with criminal legal contact, but have yet to consider the relationship between these social processes.

Legal cynicism and system avoidance are dominant features of both the penal and welfare systems, which act as interconnected forms of social control for marginalized groups. Scholars have convincingly shown how the U.S. punishment and welfare systems converge and interact as a source of both coercion and support, particularly for the poor and for Black, Native American, and immigrant communities and families (Soss et al. 2011; Rocha Beardall and Edwards 2021). Individuals engaged in multiple state systems must navigate the law and its actors in distinct, often contradictory ways (Paik 2021). Thus, people simultaneously surveilled by punishment and child welfare systems offer unique insight into how distrust and disengagement of social institutions may manifest. More specifically, the experiences of people with multi–state system involvement are important for understanding the relationship between legal cynicism and system avoidance.

Both the punishment and child support systems are often sites of state-imposed financial extraction that represent an apt site for understanding how people perceive of and engage with the law. Our study focuses on the convergence of the punishment and child welfare systems, specifically for those who owe both criminal legal debt and child support debt. We describe this situation as *dual debt* and ask: how do people navigate the experience of dual debt? How do they conceive of these systems and their relationship to them? Broadly, how do these simultaneous debts impact their lives and their plans for the future? Drawing from 30 semi-structured interviews conducted in Minnesota, we examine the material and emotional burdens of dual debt for those navigating the dictates of multiple state systems. We observe that people with dual debt routinely experience alienation from the legal system in the form of both cynicism and estrangement, which we refer to jointly as legal anomie. The design and implementation of these programs can systematically push poor people out of the formal labor market and deeper into poverty, while increasing their distrust of institutions. Through a case study of people with dual debt, we contend that legal anomie can produce system avoidance in the context of economic and social precarity (Brayne 2014). These processes are mutually reinforcing, such that legal anomie is both a response to and a catalyst for system avoidance. When experienced at a collective level, we suggest that alienating policy processes, like dual debt, may serve as mechanisms that expand legal cynicism and legal estrangement (Bell 2017; Sampson and Bartusch 1998).

## Understanding dual debt

The condition of dual debt operates at the intersection of punishment and welfare systems and the exclusionary and punitive approaches to poverty governance, or regulations and policies specifically aimed at managing impoverished populations, that animate them (Bourdieu 2004; Garland 2018; Paik 2021; Roberts 2022; Soss and Weaver 2017; Wacquant 2009). The following section contextualizes both the theoretical and empirical foundations for this paper. First, we briefly review the design, implementation, and social realities of child support and criminal legal system debt, and their intersections. These systems broadly impact already marginalized communities and reinforce social and racial inequalities. We suggest that dual debt provides a clear window into the convergence of punishment and welfare systems in the United States, or “penal-welfare hybridity” (see, e.g., Haney 2018; Sweet 2023). Finally, we define the concepts of legal anomie and system avoidance as key conceptual tools to understand the lived realities of dual debt. We contend that legal anomie and system avoidance can become deeply entangled and mutually reinforcing when law and policy design create incentive structures that penalize attachment to formal institutions.

### State-imposed debts

As state institutions have embraced neoliberal governance policies of financialization and personal responsibility, the costs associated with punishment and welfare systems have transferred from the state to the “consumer” (Brown 2017; Friedman and Pattillo 2019). These practices of “financial extraction” take a variety of forms, including fines from police contact, bail systems, “pay-to-stay” incarceration, civil asset forfeiture, and similar policies (Friedman et al. 2021; Lara-Millan 2021). These state-imposed debts have been shown to heighten economic insecurity (Harris 2016; O’Neill et al. 2022; Pleggenkuhle 2018); increase emotional and social strain in families and communities (Katzenstein and Waller 2015); and prolong criminal legal surveillance (Iratzoqui and Metcalfe 2017). Based on these findings, many sociolegal scholars argue that these kinds of state-imposed debts are a predatory form of resource extraction that exacerbate racial and economic inequalities (Harris 2020; Page and Soss 2017).

Criminal legal financial obligations are the most commonly imposed punishment in the United States, applied to offenses ranging in severity from minor traffic infractions to violent felonies. These obligations comprise a variety of monetary penalties, including fines, fees, restitution, surcharges, and other financial penalties associated with the criminal legal system (Martin et al. 2018). In 2016, 60% of people incarcerated in state prisons reported owing court costs, and 37% owed fines (Beatty and Snell 2021).

Although financial penalties may be considered a less punitive alternative to incarceration, they have significant consequences for those unable to pay them (Martin et al. 2018). These consequences include difficulty affording fundamental needs such as food, housing, and health care (Harris 2016; Pleggenkuhle 2018); greater financial stress for families and communities (Katzenstein and Waller 2015); increased stress on mental and physical health (Harris and Smith 2022); and even the loss of voting rights (Uggen et al. 2022). Nonpayment increases financial burdens by tarnishing credit, and also by triggering wage garnishment, tax-return interception, and lawsuits (Friedman and Pattillo 2019; Martin et al. 2018). Moreover, the inability to pay these obligations extends criminal legal supervision and surveillance, raising the risk of probation violations and, in many jurisdictions, incarceration for nonpayment (Harris 2016; Iratzoqui and Metcalfe 2017) in addition to other potentially criminogenic effects (Harris et al. 2010; Horowitz et al. 2022). Criminal legal financial obligations are thus not simply a penalty, but a “defining feature of a contemporary punishment regime where racial injustice is fueled by economic inequality” (Slavinski and Pettit 2021, 717).

In contrast to other forms of criminal legal debts, child support is a form of court-ordered financial support paid by noncustodial parents (parents who do not live with their children the majority of the time) either to the custodial parent directly or to the state as reimbursement for welfare services. For families that are in poverty and enrolled in public child support programs, child support payments account for an average of 41% of family income and substantially reduce child poverty rates (Turetsky and Waller 2020). Despite the popular stereotype of “deadbeat dads,” most who owe outstanding debts are unable to pay, rather than unwilling (Arthur 2018; Cammett 2010; Ha et al. 2008; Sorensen et al. 2007). An early study of noncustodial fathers and child support enforcement policies found that, for poor fathers, “the inevitable result… is the accumulation of child support arrearages, periodic jailing, and the buildup of hostility and resentment towards mothers and children as well as government authority” (Garfinkel et al. 1998, 332–33).

For impoverished noncustodial parents, child support is often a “financial bubble” that is “artificially inflated, largely uncollectible, and potentially destructive” (Brito 2019, 955). In 2021, national arrearages totaled $113 billion, a nearly 600% increase in child support arrearages since the 1980s (Haney 2022; Office of Child Support Enforcement 2021). These debts accumulate both due to nonpayment and because many states impose additional charges and interest rates of up to 12% (National Conference of State Legislatures 2021). Most of this outstanding debt is owed by people who simply cannot afford to pay it. A multistate study found that 70% of arrears, or past due payments for child support, were owed by people with less than $10,000 in annual reported income (Sorensen et al. 2007). Further, another study found that 60% of people with debt of more than $100,000 in arrears had no reported income (Arthur 2018). Consequences for nonpayment vary by state and can include suspension of professional and driver’s licenses, seizure of assets, interception of government payments and tax refunds, and even incarceration for civil contempt-of-court or criminal nonpayment.

### Penal-welfare hybridity

The penal system and the welfare system act as interconnected forms of social control for marginalized populations through both coercive and supportive programming (Bourdieu 2004; Garland 2018; Wacquant 2009), including but not limited to detention, criminal legal supervision, human and social services, and social insurance programs (Brydolf-Horwitz and Beckett 2021). Penal-welfare hybridity, the convergence and interaction of punishment and welfare systems, has been studied in settings ranging from government assistance (Headworth 2021; Simes and Tichenor 2022), prisons (Haney 2010; Sufrin 2017), policing (Stuart 2016), mandatory treatment programs (McKim 2008, 2017; McCorkel 2013; Sweet 2023), state-imposed debts (Horowitz et al. 2022; Turetsky and Waller 2020; Verma et al. 2024), and prisoner reentry (Halushka 2020; Miller 2022).

Child support and the criminal legal system represent an important site of penal-welfare hybridity. Haney (2022) estimates that the population of parents with criminal legal contact and child support orders ranges from 800,000 to 1 million individuals. Over 5 million children in the United States – or 7% – have a parent who is currently or formerly incarcerated (Murphey and Cooper 2015). A recent national analysis estimates that nearly 15% of imprisoned parents have child support orders; further, the vast majority (78%) of imprisoned parents with child support orders also have outstanding child support debt (Verma et al. 2024). In one survey of disadvantaged noncustodial parents, two-thirds of the participants had been incarcerated in jail or prison (Cancian et al. 2019).

Criminal legal involvement can negatively impact a noncustodial parent’s ability to pay child support in a number of ways. First, “the mark of a criminal record” reduces opportunities for employment, as well as wage earnings (Uggen et al. 2014; Larson et al. 2022; Pager 2003; Pager et al. 2009; Western 2002). In a survey of nearly 3,800 noncustodial fathers, over half reported that they had difficulty paying their child support due to the barriers of criminal record (Berger et al. 2021). Further, for parents with incarceration histories, child support can accumulate during incarceration, such that many parents reenter society with substantial debts (Geller et al. 2011). Formerly incarcerated noncustodial fathers accrue nearly three times more arrearages than noncustodial fathers without incarceration histories (Emory et al. 2020). Parents with criminal records carry the dual burdens of accumulated child support debt and the stigma of criminal legal system involvement. In addition, the financial obligations that often accompany imprisonment contribute to these debts. Formerly incarcerated men with child support debt are criminalized through punitive civil enforcement measures and feedback loops of disadvantage, a process Haney (2022) defines as an “imprisonment of debt.” Through penalties for noncompliance and the resulting survival strategies that people adopt in response, Haney (2022, 156) argues, the system “produces exactly the kind of subjects it most fears: men who retreat underground, violate rules, and commit crimes.”

Horowitz et al. (2022) find that individuals holding debt in both the criminal legal and child support systems owed significantly higher debt amounts in both systems, compared to individuals who owed debt in only one system; this finding suggests these debts may be conditional on one another and are not merely additive but compounding. Further, such debts are unevenly distributed, with Black and Native American men owing greater amounts than white men (Horowitz et al. 2022). Such work highlights the particular vulnerability of people who are dually indebted, as well as potential mechanisms connecting dual debt status to perceptions regarding the legitimacy of these systems (Horowitz et al. 2022).

### Legal anomie

We suggest that the policy design of criminal legal and child support debt, and the experience of owing significant debts to multiple punitive systems create fertile ground for the development of legal anomie. We follow classic Durkheimian theory and define legal anomie as a state of normlessness that results from broken social bonds and social disorder via the law and legal authorities (Durkheim 1979 [1897]). Beyond negativity, anomie is a state of alienation, or “a sense that the very fabric of the social world is in chaos – a sense of social estrangement, meaninglessness, and powerlessness, often a result of structural instability and social change” (Bell 2017, 2085). The collective experience of legal anomie, when discrete social groups or social geographies experience alienation from legal systems, can result in legal cynicism and legal estrangement.

Legal cynicism^1^ is a negative, even hopeless framing of the law, its legitimacy, and the ability of those who work within the legal system to do so in an effective nondiscriminatory manner (Sampson and Bartusch 1998). Bell (2017) extends the concept of legal cynicism with legal estrangement,^2^ or a theory of alienation from the law’s enforcers that “reflects the intuition among many people in poor communities of color that the law operates to exclude them from society” (2017:2054). Whereas much legal cynicism research has emphasized neighborhood differences, legal estrangement foregrounds macro-structural processes rooted in race-class subjugation and the maintenance of “concentrated poverty and racial inequality” (2126). While legal cynicism and legal estrangement are collective processes, our analysis focuses on individual experiences with the law and legal systems. We suggest that when legal systems produce anomic conditions among individuals that aggregate to social groups, they can produce or contribute to social realities of legal cynicism and legal estrangement.

### System avoidance

Interactions with legal systems and actors not only influence individuals’ perceptions of the law but also impact their willingness to engage with other social institutions. Brayne (2014) finds that individuals engaged with the criminal legal system – from police stops to incarceration – are less likely to engage with other record-keeping institutions, such as educational, medical, financial, and labor systems. She proposes a theory of system avoidance, which refers to the process of dissociating and disconnecting from social institutions that keep formal records in order to reduce surveillance (Brayne 2014). By further disconnecting an already marginalized group further from society, these avoidant practices can worsen pre-existing inequalities. System avoidance takes on new meaning in the digital age in which criminal records are easily accessible and managing the stigma of criminality is increasingly difficult (Lageson 2020; Lageson and Maruna 2018). Such system avoidance has been documented in a broad array of policy arenas that employ surveillance techniques (Abrego and Menjívar 2011; Asad 2023; Yoshikawa 2011).

Tactics of system avoidance are particularly salient for parents under state systems of family control. For example, for mothers navigating Child Protective Services (CPS), system avoidance can be impractical or even counterproductive. Instead, mothers engage in “selective visibility,” participating in state systems while obfuscating hardships or vulnerabilities that may invoke state responses (Fong 2019). Similarly, undocumented immigrant parents seek state inclusion via public assistance to support their children, even though such engagement can lead to unwanted state involvement, including CPS investigations, loss of parental rights, or even deportation (Asad 2023). Formerly incarcerated Black women engage in similar tactics of selective visibility to navigate both carceral and familial forms of state control, even as they identify the state and the possibility of child removal as the greatest danger to their children (Gurusami 2019). Gendered differences in system avoidance of policing are largely explained by parenthood and specifically, motherhood, rather than gender alone (Ascherio 2023). Therefore, parenthood and state forms of family control often necessitate distinct strategies of system engagement, rather than strict avoidance.

Recent research suggests that fathers are more likely to avoid formal institutions, or engage in “partial system avoidance.” Haney (2022) finds that fathers paying child support may combine jobs in the formal labor market to pay debts via wage garnishment while supplementing their income with informal opportunities. She argues that fathers engage in system avoidance “to avoid state control, not to avoid supporting their kids” (165). However, these strategies often backfire, in part because they can limit fathers’ access to the state benefits that provide needed support to financially precarious men. Some research suggests that system avoidance is a bifurcated and raced phenomenon, finding that criminal legal contact is associated with decreased involvement in medical, economic, and sometimes religious institutions but greater involvement in activism and, for Black men, volunteering (Remster and Kramer 2018). Formerly incarcerated fathers (though not formerly incarcerated mothers) are generally less likely to participate in school activities with their children after incarceration (Haskins and Jacobsen 2017). Thus, strategies of system avoidance are broadly observed for populations with criminal legal contact, although such strategies are clearly influenced by other identities, including gender and parenthood.

### Conceptual model: the cyclical process of dual debt, legal anomie, and system avoidance

**Figure 1.**
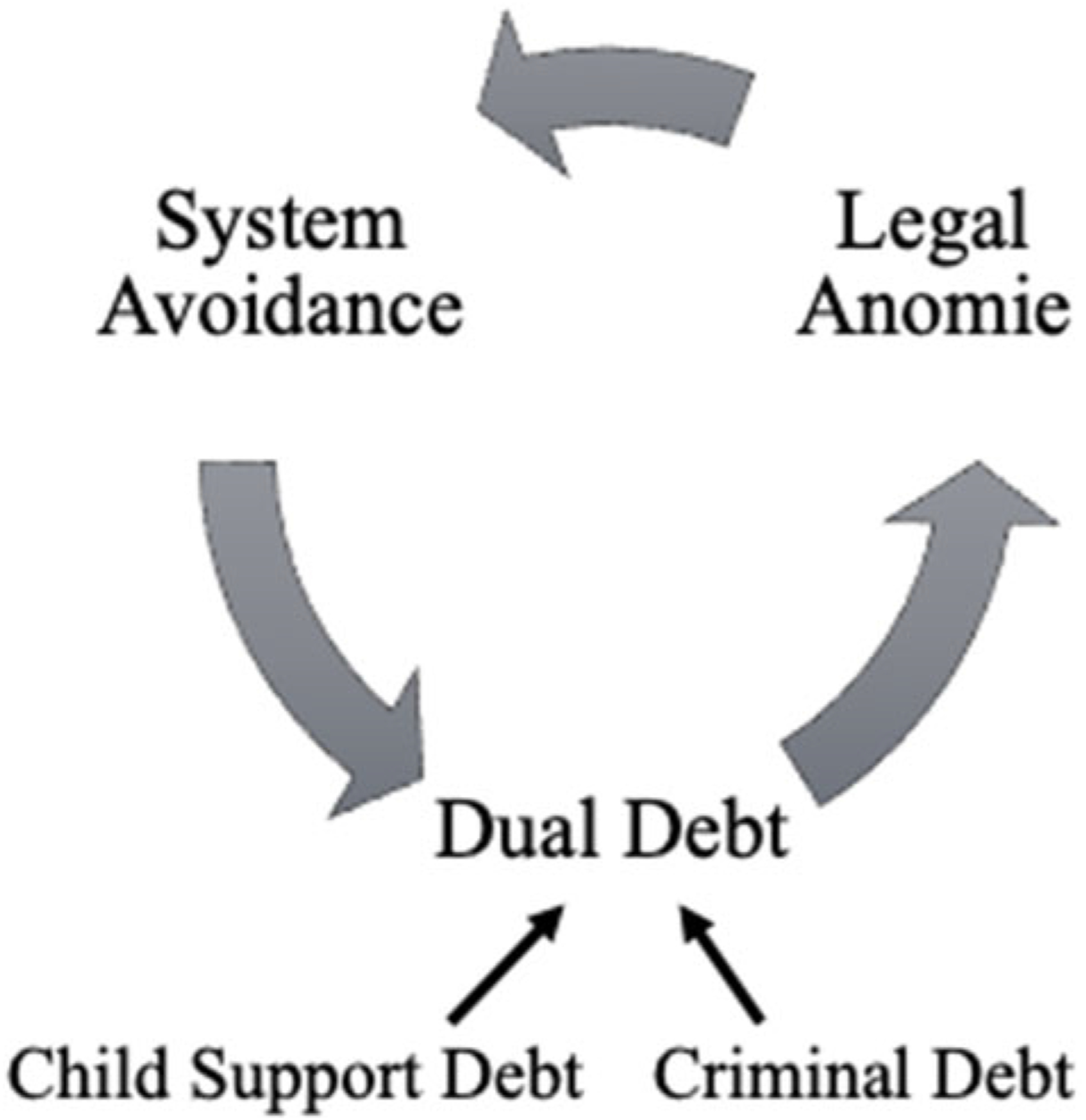
An integrated conceptual model of dual debt, legal anomie, and system avoidance.

Drawing from this literature, we suggest a mutually reinforcing relationship between legal anomie and system avoidance, described in Figure 1. The imposition of significant legal debts with punitive surveillance and collections mechanisms creates conditions of legal anomie, and directly incentivizes system avoidance for people with dual debt. By engaging in system avoidance, however, people with dual debt often accrue penalties and greater debt, further alienating them from both legal systems and core social institutions like the formal labor market. This alienation increases system avoidance, resulting in a compounding cyclical process.

System avoidance can thus be shaped and incentivized by policy design. Stigmatization that pushes people out, coupled with policy designs that penalize contact with record-keeping institutions, increases the incentives for system avoidance. System-avoidant behaviors can then create a cascade of consequences that accelerate and compound legal anomie. As legal anomie becomes entrenched, system avoidance becomes routine and morally acceptable. When experienced at a collective level, these policy systems heighten legal cynicism and legal estrangement by expanding legal anomie across neighborhoods and race-class subjugated communities.

We argue that legal anomie and system avoidance are mutually reinforcing processes above and beyond their shared roots in structural social exclusion. Under conditions of alienation from core social institutions, including the labor market and the housing market, normative pressures to remain attached to ordinary legal processes are diminished. Systems of family control and criminallegal control engender different forms of exclusion and hence different types of system avoidance. For the dual debt population that experiences these systems in combination, we anticipate observing both legal anomie and system avoidance, due in part to policy designs that directly deter continued attachment to formal institutions and foster alienation.

## Data and methods

Our study is situated in Minnesota, which is neither a high-arrears state nor a comparatively punitive state in its policies for child support debt or criminal legal financial obligations (Harris et al. 2017).^3^ There is significant state-to-state variation in child support and Minnesota has a higher-than-average percentage of imprisoned parents with child support debt (Verma et al. 2024). However, Minnesota’s system of monetary sanctions is less severe than other states in terms of both the amounts ordered and the consequences for nonpayment. For example, a conviction for driving with a suspended license garners a range of imposed criminallegal financial obligations from $585 to $735 in Wisconsin, as compared to $279.50 to $289.50 in Minnesota (Harris 2016; Harris et al. 2017).

Although Minnesota’s systems of child support and monetary sanctions are generally considered progressive relative to other U.S. states, the consequences for nonpayment include driver’s license suspensions, probation violations, tax-fund interceptions, as well as late fees and additional costs (Sec. 171.16 MN Statutes 2023; Sec. 270A.10 MN Statutes 2023; Sec. 480.15 MN Statutes 2023; Sec. 518A.65 MN Statutes 2023). For those living outside of metropolitan areas, the loss of a driver’s license (which can result from both unpaid legal financial obligations and unpaid child support) may be especially consequential. While Minnesota law prohibits incarceration and extended probation for unpaid monetary sanctions (Constitution of the State of Minnesota Article 1, Section 12. 1857 ), it allows probation to be extended for unpaid restitution (Sec. 609.104 MN Statutes 2023). Although Minnesota has a unified court system to maintain consistency across the state, in practice both monetary sanction amounts and required fees vary widely across municipalities (Harris et al. 2017). Eventually, unpaid criminal debt owed to the courts is sent to Minnesota’s revenue recapture (Sec. 270A.07 MN Statutes 2023). When this occurs, a 20% fee is added to the debt. Revenue recapture, which operates as the sole debt collector for the state, collects outstanding criminallegal debts through tax-refund intercept rather than wage garnishments. In contrast, child support orders and arrears (Sec. 571.922 MN Statutes 2023) are often collected through wage garnishment and in some circumstances can result in up to 65% of disposable income being garnished.^4^ In addition, outstanding child support debt is also collected by Minnesota’s revenue recapture program, taking priority over unpaid restitution and court debts (Sec. 270A.10 MN Statutes 2023).

Our analysis draws on data from 30 semi-structured interviews with Minnesota residents subject to both child support and criminal legal debt. Our interviews were conducted between September 2018 and March 2020.^5^ Participants were recruited through purposive sampling based on online recruitment of people with dual debt obligations (Craigslist or Facebook) and through connections with community partners that serve a high proportion of clients with dual debt obligations, such as a faith-based substance use treatment programs.

It is important to note that our interview sample reflects the state’s racial demographics: Minnesota has a whiter population than many other states. Consequently, our data offer limited insights into the experiences of people of color and specifically, race-class subjugated communities. Instead, our study examines a racial-majority but class-subjugated population and considers the extent to which elements of legal cynicism, estrangement, anomie, and system avoidance are observed in this majority-white sample.

Our interview guide included semi-structured questions designed to help us understand the firsthand experiences of persons subject to dual debts (see Appendix A). We asked respondents open-ended questions about their engagement with the child support and criminal legal systems separately and in combination. The interviews ranged in duration from 45 minutes to over 2 hours. All interviews were transcribed, organized, and coded with NVivo software. The research team developed a coding scheme based on preliminary analysis of the interviews, considering insights derived from existing theoretical frameworks regarding the criminal legal and child support systems (i.e., Haney 2022; Horowitz et al. 2022). All transcripts were first coded into broad, theoretically grounded categories such as system avoidance, legal cynicism, and criticism. In the initial round of coding “legal cynicism and criticism” were a single-coded item. After an initial round of coding, subcodes were developed (i.e., legal cynicism/criticism was recoded into “never-ending or overpowering debts,” “poverty penalties/interest,” and “critiques of specific fees”). We include a table of these subcodes and their prevalence in Appendix B. Although many of the subcodes we identified were manifestations of legal anomie or cynicism, a few subcodes did not fit as well within this framework.^6^ Next, we analyzed our data using the NVivo query features with attention to which patterns of codes were strongest (most prevalent) and how these codes overlapped (or failed to overlap) with one another within interviews and within blocks of text.

## Results

### The experience of dual debt

Our interviewees were primarily men (83%), consistent with the gender distribution of noncustodial parents (Grall 2020). The interview sample was also demographically similar to the state population of people with dual debt (Horowitz et al. 2022). Approximately 60% self-identified as white, followed by approximately 17% Black and 17% Native American. Our participants had between one and eight children and between one and three child support orders. Less than half (43%) were employed in the formal labor market at the time of our interviews. Half of the interviewees had been convicted of a felony and 37% had been convicted of solely a misdemeanor. Our participants were more knowledgeable about their child support debt amounts than criminal legal debt amounts, and child support debts were much larger than criminal legal debts. One third of our participants did not know how much they owed in criminal legal debts. For those who did know, they reported owing from $0 to $9,000. Four of our participants had paid off their criminal legal debts. In contrast, only one participant reported having paid off his child support debt. The other participants reported owing between $1,200 and $130,000 in child support arrears.

Although child support debt amounts tended to be greater and more salient than criminal legal debts for our participants, the two were experienced in tandem. Holding criminal legal and child support debts simultaneously placed our respondents in a legally and financially difficult position. When discussing the criminal legal system and the child support system, Jared, a 43-year-old white man, found the joint impact of the debts particularly onerous. He explained how these two systems interacted:

It’s a traffic ticket. But in order to get my license back, I have to agree to exorbitant fees, and pay down amounts. ‘Okay, if you want your license back you have to pay us all this extra,’ because they hold all the cards. There’s no place else I can go. Okay, I’ll agree to that, okay, fine, give me my license back. So they added on a whole bunch of stuff to my child support, which puts me further behind, so I can still go out to work and to eat. So it’s *two separate systems that are working together to kind of keep you under a thumb*. (Interview, March 14, 2019, emphasis added)

Jared couldn’t afford to pay his traffic ticket, which he received because he had no car insurance – another expense beyond his means. Because he was unable to pay the initial ticket, he lost his driver’s license (a consequence of nonpayment at the time) and was also charged additional fees. Jared risked additional criminal charges for driving to his job without a license. But if he had not worked, he would have fallen behind on the child support payments garnished from his wages, along with other essential expenses. Even if he had already paid his traffic ticket and fees to reinstate his license, child support enforcement can suspend his license if his outstanding child support debt increases too much (Sec. 518A.65 MN Statutes 2023). Meanwhile, such child support debts are accumulating and accruing interest. Thus, Jared’s difficulty getting to work due to the traffic ticket put him at risk of additional penalties not only from the criminal courts, but also from child support enforcement. Jared’s story highlights several key themes: the loss of a driver’s license as a disruption to employment, the additional obstacles tied to penalties for nonpayment, and the difficulty or inability to pay fines along with child support. Here the combination of criminal legal involvement, even at a misdemeanor level, along with outstanding child support payments, posed distinct financial and legal challenges.

The consequences of nonpayment presented additional barriers to paying off debts and meeting essential expenses. Like Jared, many other participants highlighted how losing their driver’s license due to unpaid criminal legal debt impacted their employment and finances. William, a 47-year-old formerly incarcerated Native American man, lost his job when his license was suspended because he could no longer operate heavy machinery (Interview, October 14, 2018). He shared his frustrations over losing this good job: “I just don’t understand why they would do that because if you can’t drive, you can’t get to the good jobs, how are you supposed to pay your bills?” William worried about the cascading effects of these difficulties. “It’s on my mind all the time,” he said, “how the hell am I going to get out of this money and get it paid off and get my child support paid off and my license back, pay rent, pay bills, keep the cars running, keep everybody happy?”

The stigma of a felony record raised additional barriers for people with dual debt. Participants discussed their difficulty finding work, a common issue for people with criminallegal involvement (Pager 2003). Jeremy, a 30-year-old formerly incarcerated white man who owed over $55,000 in child support arrears, noted how his felony record impacted his job prospects and the ensuing financial consequences:

For a guy like me … most [of] the times that I’ve been incarcerated is because of mental health or drug use. For my entire life and my entire future to be ruined. I can’t get a job at certain places. My face and my picture’s all over the news and stuff like that. I don’t think that’s right. Then you get in all this debt and then it affects your credit. (Interview, October 5, 2018)

People fall even further behind in their debt payments during periods of incarceration, sometimes without their knowledge. Lee, a 53-year-old Black man, explained that during his 3-year incarceration, he was unaware that his child support debts continued to accumulate (Interview, September 14, 2018). Such payments were impossible to make on low- or no-wage prison jobs: “How are you charging me five hundred and something dollars a month, and I make 12 cents an hour?” At a rate of 12 cents per hour, Lee would have to work 4,167 hours per month (or 137 hours per day) to make his $500 monthly payment. People with other cases or other forms of criminal legal involvement – such as traffic tickets, misdemeanor fines, and probation – experienced additional burdens to paying both child support and criminal legal debts. Notably, such systems often fail to consider one another when assessing sanctions, child support calculations do not typically consider outstanding criminal legal debt, and judges may not take child support obligations into account when imposing monetary sanctions.

Other financial consequences also flowed from child support and criminal legal debts, including reduced credit scores, related difficulties receiving loans or credit cards, and seizure of tax refunds and other benefits. Adam, a 36-year-old white man, shared how his life would be different without these two sources of debt:

Well, it would be totally different, because I wouldn’t have to focus completely on the law, or worry about if I miss a payment, am I going to lose my license? Am I going to go to jail?^7^ Well and then I don’t have any liens against me and a bank would be more willing to give me a loan, and you know, credit cards [companies] would be more willing to give me a credit card. (Interview, September 14, 2019)

Adam highlights how these two overlapping debts produce distinct repercussions, including not only potential criminal legal contact but also financial consequences. As people with dual debt demonstrate, different kinds of debts are perceived and experienced based on their moral valence (Greenberg et al. 2020). While some debts are considered “good” to consumers, such as mortgages or student loans, other debts are labeled bad and thus are more stigmatized, such as credit card debt (Peñaloza and Barnhart 2011) and court debt (Horowitz et al. 2024). Such distinctions influence financial decisions about how to prioritize different debts and impact financial health (Greenberg and Hershfield 2019). Further, the accumulation of debt significantly impacts perceptions of personal wealth (Sussman and Shafir 2012) while also producing emotional distress (Hershfield and Greenberg 2016). Previous research on individuals with dual debt suggests that child support debt is often more salient than criminal legal debt due to the higher amounts, regular notices, and wage garnishment policies (Horowitz et al. 2022). In general, people with dual debt prioritize their child support debt when making financial decisions because it has a greater impact on their daily lives. Nevertheless, the combined financial difficulties of child support debt and criminal legal debt, along with the stigma of criminal legal contact, produce particular financial burdens and legal consequences that impact decision making. In short, participants described the experience of dual debt as a “vicious cycle” that reverberates in their everyday lives, including their employment, finances, and wellbeing (Interview with a 30-year-old white man, October 5, 2018).

### Legal anomie

Interviewees overwhelmingly characterized both the civil and criminal legal systems as unfair, harmful, and illegitimate. We understand these individual perspectives as an expression of legal anomie, a state of normlessness resulting from broken social bonds and social disorder via the law and legal authorities. These frames were especially salient as people discussed their outstanding debt amounts and the associated legal and financial consequences. Participants found that the sanctions for nonpayment, such as wage garnishment and driver’s license suspensions, were counterproductive to the goal of paying their debt, and prolonged their involvement in the system. Some interviewees also suggested or explicitly labeled state agencies as corrupt entities profiting off debt collection. Taken collectively, these individual experiences of legal anomie suggest that people entangled in both the civil and criminal legal systems broadly experience legal cynicism and legal estrangement.

People with dual debt described both the child support and criminal legal systems as institutionally “broken programs” that undermined and confounded them. Devin, a 40-year-old formerly incarcerated white man, described them as “good programs, but I think they’re all broken programs, as far as how they work, because they don’t actually do what they’re intended to do” (Interview, October 4, 2018). While individuals might agree with the principles of the criminal legal system or the child support system, their experience led them to see their actual processes as harmful. Consistently, interviewees saw the systems as counterproductive because the consequences for nonpayment hindered their ability to pay outstanding debts. These overwhelmingly negative perceptions were intensified by experiences navigating the consequences of noncompliance, including wage garnishment, driver’s license suspensions, and interceptions of tax refunds. For example, Nate, a 33-year-old Black man, understood the importance of child support but said the system made it difficult to “make a living,” explaining: “[Y]ou should just have your money taken away from you. Not your ways to make money. That seems backwards” (Interview, September 14, 2019). Others connected their cynicism to the great stress they felt from owing large sums. Geoff, a 32-year-old Native American man, viewed his debt as so impossible to settle that the specific amount owed was no longer relevant: “It might as well be a million dollars … it doesn’t matter how much it is. It’s too much. Too much is too much. That definitely seems like they just want you in debt your entire life” (Interview, March 17, 2019). Geoff links the burden of seemingly endless indebtedness to both the scale of his outstanding debt and the perceived ill intent of the legal systems that imposed it.

Such sentiments extended to both the criminal legal and child support systems. Carl, a 52-year-old formerly incarcerated Black man, said the systems were similar because “[b]oth of them is like roadblocks. They make you feel like you don’t have no way out. You can’t grow. You can’t progress with that in the way” (Interview, October 14, 2018). These roadblocks impacted participants’ capacity to plan for the future. As Jared explained, “For me to make a plan is for me to hang myself with a rope of hope … I am stuck in their hole until I work myself out of it.” Some considered these systems as not only counterproductive, but predatory or even criminal. Danny, a 58-year-old Native American man, compared his experiences with the child support system to the mob: “To me, it’s like, man, these guys [the child support system] are just like criminals, mobsters or something.… They want to swoop on your loot. They make taxes for everything. They’ll swoop on everything.” Danny considered the repeated interference of the child support system with his earnings as akin to corruption, likening his legal debts to “taxes.”

In short, people with dual debt consistently experienced a state of legal anomie where they understood both civil and criminal law as broken, illegitimate, and alienating. Study participants expressed deep frustration with the design and implementation of the child support and criminal legal systems, even as they articulated the potential value of such systems. They perceived enforcement mechanisms attached to criminal legal and child support debt to be counterproductive, particularly regarding integration into the labor market and ordinary social life. This tough enforcement, combined with high debt amounts, led participants to see these systems as fundamentally unfair and unreasonable. To the extent that they perceived these debts as permanent and insurmountable, they also articulated a sense of despair.

### System avoidance

System avoidance was a routine practice for people with dual debt in our study. In 20 of 30 interviews, participants described specific strategies to avoid record-keeping institutions. For example, they took “cash jobs” to avoid credit cards, bank accounts, taxes, or accumulating further debt. People with dual debt also avoided financial institutions by “paying cash for everything” (Interview with a 53-year-old Black man, September 14, 2018). Because of this tactic, many participants had poor credit or no credit, which hindered their efforts to rent homes, buy vehicles, and obtain loans or credit cards. Other types of system avoidance included transferring assets to avoid their garnishment or seizure from financial institutions (Sec. 518.68 MN Statutes 2023). After a coworker’s bank account was unexpectedly emptied by child support enforcement, Jeremy cut ties with banks: “I don’t put anything in my name because I’m scared they’re going to take it. Bank accounts, vehicles, anything like that” (Interview, October 5, 2018). By staying “off the grid,” Jeremy sought to protect his limited assets from being seized by state agencies (Interview with a 58-year-old Hispanic man, October 5, 2018).

Participants also engaged in informal work or cash jobs as a means to supplement their income. Ken, a 38-year-old formerly incarcerated white man, explained the necessity of informal earning opportunities: “If I want to survive, and pay bills, I have to. Because if I go get a 9 to 5 job and do the paychecks, I’m not going to make enough to pay bills” (Interview, October 4, 2018). Due to the high rate of wage garnishment for child support arrears, Ken could not afford essential expenses, such as rent, food, and electricity, without “off the grid” income. Jerome, a 31-year-old Black man, also noted that formal work would be insufficient: “I mean if I just worked my jobs with stuff that I got going on legally, it’s like dude, I’d be homeless. So, it’s like I got to hustle too” (Interview, December 7, 2020). System avoidance was therefore both a response to legal anomie and a strategy for maintaining a minimal standard of living.

Although these strategies were primarily used to manage the consequences of noncompliance for child support debt, we note that people with dual debt often carry heavier child support debt *because* of their multiple-system involvement. As noted above, individuals with dual debt owe significantly more in both the child support and criminallegal systems, compared to those owing debt in only one system; this suggests these debts may be conditional on one another and are not merely additive but compounding (Horowitz et al. 2022).

Some participants not only avoided making payments by leaving the formal labor market, they also ignored *information* about the payments – a subtheme that emerged organically in our data collection that could be explored in future research. For example, monthly statements were such a source of stress that some elected to “throw [them] in the trash” (Interview with a 32-year-old Native American man, March 17, 2019). Jeremy, a 30-year-old white man, explained his response to monthly statements: “I see how many tens of thousands of dollars that I’m behind, and it’s just discouraging, man. I’m trying to build a family somewhere, and get out of debt, stay out of prison. Owing that kind of money is crazy” (Interview, October 5, 2018). Jeremy viewed his debt as a barrier to becoming a financially stable, law-abiding parent, and the associated stress led him to avoid information from child support agencies. Similarly, Geoff also viewed this information as a source of significant stress:

When you’re trying to survive, rent, food, all that.… Are you really trying to pursue knowing the information about what you owe? I feel like knowing it is hard. Just knowing it is a hard pill to swallow because … if you can’t do anything about it, worrying about it, that’s really hard. It adds to other worries that you already have that are so major. (Interview with a 32-year-old Native American man, March 17, 2019)

Geoff’s monthly statements reminded him of his inability to settle outstanding debts, which produced further stress. Because he was already struggling to afford essentials like rent and food, the specter of his outstanding debts was more than he could bear. Jeremy and Geoff expressed hopelessness in response to circumstances they saw as insurmountable. As with other forms of system avoidance, these avoidant strategies are likely rooted in economic and social precarity.

Jeremy and Geoff thus managed the emotional stress of their debts through “information avoidance,” a well-documented concept in psychology. Information avoidance is “any behavior intended to prevent or delay the acquisition of available but potentially unwanted information” (Sweeny et al. 2010, 341). People engage in information avoidance to maintain a belief, avoid undesired outcomes, or manage undesired emotions (Sweeny et al. 2010). Strategies of information avoidance are impacted by perceptions of risk, coping mechanisms, and social norms, including trust in institutions (Foust and Taber 2023). We consider this an important area of inquiry within the study of system avoidance with potentially important implications for individuals with multisystem involvement. Understanding information avoidance as an extension of system avoidance may also prove useful in understanding other court processes.^8^

### Legal anomie and system avoidance

We observed distinct patterns of legal anomie and system avoidance throughout our interviews, but we also saw how these processes overlapped, particularly as participants discussed the harsh consequences of nonpayment. In moments where distrust in and disengagement from formal institutions overlapped in interviews, participants often discussed the material hardship that stemmed from their debt payments in combination with other financial stressors and perceived inequities in the systems themselves. When the size of their debt was so great and the penalties for nonpayment so steep, people with dual debt felt powerless, trapped between the harsh penalties of noncompliance and their inability to pay. This dejection was heightened by substantial wage garnishment, which left some participants with little or no earnings. Such participants often rejected the formal labor market and legal system altogether. Based on the experiences of people with dual debt, we contend that legal anomie and system avoidance are interrelated and mutually reinforcing social processes, especially for those living in poverty. This process is important for understanding how legal cynicism and legal estrangement manifest in race-class subjugated communities.

Lisa, a 47-year-old formerly incarcerated white woman, shared how the confluence of child support debt and criminal legal involvement felt so dehumanizing that people only saw her for her debt and felony record:

They’re setting me up for failure before I even walk out of that courtroom. I was in a treatment center to get well and they’re racking up the child support… I mean, it doesn’t make sense to me. It makes me so frustrated I wanna cry, which I do every single day. If I’m not crying because I ran out of something, I’m crying because I have so much debt, or I’m crying because in a year and a half I’m gonna have to find if I have a place to live, and a job, and a way to support myself.… I feel like *my right arm should be ‘debt’ and my left arm should be ‘felony’, ‘cause that’s what everybody sees when they look at me*. That’s all that I am. When I’m trying to get a car, or an apartment, or whatever. And when I call and try and pay any of my debt off, they talk to me like I’m a piece of crap. (Interview, March 13, 2019, emphasis added).

Lisa experienced such profound alienation from the legal system that she no longer felt human. While undergoing court-ordered drug treatment, her child support debt continued to mount. She felt “set up for failure” because she is subject to two conflicting mandates: complete inpatient treatment and keep up with her child support payments. As she attempted to cover essential expenses, she continually faced barriers due to her debt and felony status. This process produced significant emotional distress, along with the material impossibility of paying off her debts.

Bourdieu (2004) famously distinguished between the “right” and “left” hands of the modern state, with the right representing banking and finance and the left representing social services. Wacquant extended this distinction (2009) by situating the state’s “criminal justice arm” on the right. Lisa uses a related metaphor in describing her situation, putting “debt” on her right arm and “felony” on her left. Perhaps tellingly, she makes no mention of social services or support.

Seemingly insurmountable debt amounts, along with high rates of wage garnishment, prompted some participants to reject the child support system or even the legal system altogether. Ken, a 38-year-old white man, explains his cynicism about program rules and his choice to engage in system avoidance:

They can take up to 60% in the state of Minnesota.… Well, I can’t survive on that. So why give them money? And on top of it, why give them money when I still take care of my child?.. I don’t feel that I should bust my butt to pay child support when they’re not working with me. I mean, $23,000. I’ll never pay that off. (Interview, October 4, 2018)

Ken’s assertion that the state could garner up to 60% of his wages is consistent with Minnesota law (Sec. 571.922 MN Statutes 2023). He simply could not see how he could maintain a basic living on less than half his paycheck. Unable to pay his debts or survive on his garnished wages, Ken responded by removing himself from the formal labor market and refusing to pay his child support debt.

Many interviewees shared similar experiences of impoverishment due to high wage garnishment. Lee, a 53-year-old Black man, discussed leaving the formal labor market after garnishment for child support debt left him with less than $30 in his paycheck:

I’ll never forget, I went to work, and … my paycheck was $29.16 …That’s all they left me with, that wasn’t even enough to buy gas to get to work for the next week, and that’s all they left me with. I was just like, ‘You gotta be kidding.’ After that encounter happened, that’s when I went underground.

Later in the interview, Lee explained further:

Because it’s like, ‘Okay, I’ve paid my child support bill, I paid this, I paid that, but I still got $75 bucks left over, okay, that’s my money.’ But when you leave with nothing, that breeds ugliness, I can’t get the right word, but you know what I mean? It breeds animosity and things, you get to that point, ‘Well, I’m not paying nothing.’ (Interview, September 14, 2018)

Like Ken, Lee’s experience of being left “with nothing” contributed to his rejection of both the child support system and the formal labor market. We view Lee’s references to “ugliness” and “animosity” as expressions of legal anomie, which directly contributed to his decision to avoid social institutions. The process of debt collection produces such intense impoverishment that it pushes him “underground.” This response coincides with previous research showing that compliance with child support payments declines once orders exceed 30% of an individual’s earnings (Hodges et al. 2020).

Such accounts show how legal anomie and system avoidance are mutually reinforcing as participants grapple with their debts. Consistent with the process described in Figure 1, we observe a pattern in which the policies for collecting outstanding civil and criminal debts foster alienation from the legal system, or legal anomie. As people with dual debt face both legal and financial consequences, they disengage from the law and the formal labor market. Such strategies of system avoidance support their short-term needs, but increase their debts, making them more susceptible to continued surveillance and nonpayment consequences. In turn, such consequences heighten legal anomie, resulting in a compounding cyclical process, particularly for those in under-resourced communities.

Other participants forewent essential items when wage garnishment removed a substantial portion of their already low earnings. Nathan, a 43-year-old formerly incarcerated biracial man, had been paying off his child support arrears while experiencing homelessness, then left his job:

I would’ve gotten maybe like $140 at each check to live off for two weeks. And I was homeless. I just quit my job. I just went back to the streets, there’s no way I’m going to let people just take my money, and I’m not going to work this job and only have $140 because it’s just going to make me hopeless, even more hopeless than I am. So that’s when I basically stopped working legitimate jobs. (Interview, October 14, 2018)

Despite working a full-time job, Nathan’s earnings were so low after wage garnishment that he was unable to afford housing or other necessities. This deprivation, along with the mental and emotional stress it produced, ultimately led him to disengage and abandon the formal labor market. Over time, such strategies only increase debts and put individuals at risk for further sanctions. Here too, we observed a cyclical process in which expressions of legal anomie and system avoidance are co-occurring, prompted by alienating policy designs that push people out of formal institutions. Such findings echo Haney’s (2022) conception of the “imprisonment of debt,” in which debt and the associated penalties undermine efforts to attain financial stability and break free of the surveillance of state agencies.

Nathan also explained how his firsthand experience with “how the system is run” left him disillusioned:

It just follows you around and you get desensitized to it, where people think you don’t care, it’s not that you don’t care, it’s just when you see how the system is run when you’re actually in it, and then you realize that *the people that are writing these rules don’t follow these rules that don’t apply to them* (emphasis added). Because it’s just that mental stress of that, it’s a lot. They think that people don’t care, and it’s not that. What can you do about it if you ain’t got no money and you got a drug habit and your family’s falling apart and everything’s going bad and you ain’t got nowhere to turn? So you get hopeless. (Interview, October 14, 2018)

Nathan clearly articulates a state of legal anomie in which rules are unfairly applied and legal actors cannot be trusted. Unable to pay his debts, he feels “desensitized” and alienated from a dysfunctional legal process. Further, his debts only added to the significant stresses of poverty, addiction, and broken family ties, increasing his economic insecurity. Nathan can neither improve his economic or social position, nor remove himself from the constraints of the punishment and child welfare institutions to which he is indebted, leaving him feeling hopeless. This experience of being entangled within an alienating system was both materially and psychologically damaging for participants who were unable to pay off their combined debts.

## Discussion and conclusion

We have argued that experiencing dual debt can heighten legal anomie, or a state of profound alienation from and distrust of legal institutions (Durkheim 1979 [1897]). People holding criminal legal and child support debt are subject to law and policy that penalizes involvement in the formal labor market, exposes them to discrimination, and renders routine social participation more difficult. We describe these features of dual debt policy design as alienating, in that they push individuals out of participation in core social institutions. That is, people with dual debt, especially those already living in poverty, experience greater precarity as opportunities for subsistence through employment or access to welfare state programs are closed off. This engenders greater distrust of the law and its institutions while simultaneously exerting pressure to seek alternative means of survival.

We show that many people with dual debt follow a common pattern toward system avoidance. They are stigmatized by the visible negative credential of criminal legal system involvement (Lageson 2020), which negatively impacts employment and earnings in the formal labor market (Uggen et al. 2014; Pager 2003). At the same time, child support debt (and to a lesser extent criminal legal debt) routinely results in court orders to garnish wages from formal employment. Because criminal records often relegate workers to the lower rungs of the formal economy (e.g., Western 2002), people with dual debt routinely see garnishments capture up to 60% of their paychecks. When debt loads are so high, they face strong pressures to exit the formal labor market and seek informal income. In this way, criminal record stigma and mechanisms of debt repayment interact to pattern experiences with, and withdrawal from, formal institutions. People with dual debt routinely describe their challenges as insurmountable, oppressive, and unjust, while articulating personal narratives of exclusion and cynical orientations toward legal systems. Many destitute participants reject the formal labor market in lieu of informal earning opportunities to meet immediate material needs. Although such strategies may offer short-term benefits, they further entangle people in greater debt and further marginalize them from important social institutions. In sum, the interactions of stigma, labor market discrimination, large legal debt loads, and wage garnishment systematically channel people with dual debt into routine practices of system avoidance and legal anomie, highlighting injustice in both criminal legal and child support institutions.

At the core of this process is a fundamental contradiction: people with dual debt are incentivized to avoid the institutions that could help them pay their debts and reestablish beneficial social relationships. As they disengaged from the formal labor market, those with the most onerous debt loads routinely sought off-the-books work as a survival strategy. And these system-avoidant behaviors began a cascade of processes that further disconnected participants and legal institutions. Nearly all of our participants expressed profound distrust of legal systems coupled with avoidance from formal institutions, leaving them in a condition of legal anomie. As we saw in our results, particularly for participants such as Lee and Nathan, these multiple state-imposed debts and their associated consequences are experienced collectively. This should not be surprising when, in many jurisdictions, the two institutions are housed in the same county building. To better understand the situation of those simultaneously involved in the child support and criminal legal system, we should therefore conceive of and study such processes in the same way they are experienced.

Additionally, we argue that exclusionary policy designs, including current debt collection processes in both the child support and criminal legal system incentivize avoidance. Particularly for the poor, these policy designs may necessitate system avoidance as a survival strategy. In doing so, these systems can create and reinforce legal anomie. When these processes are coupled with aggressive racialized policing and longstanding structural disadvantage, it is likely that they engender legal cynicism and, for some, legal estrangement. We therefore contend that legal anomie and system avoidance are mutually reinforcing processes above and beyond their shared roots in structural social exclusion. Under conditions of alienation from core social institutions, including the labor market, normative pressures to remain attached to ordinary legal processes are diminished. Systems of family control and criminallegal control engender different forms of exclusion and hence different types of system avoidance. For the dual debt population that experiences these systems in combination, we observe both legal anomie and system avoidance, due in part to policy designs that directly deter continued attachment to formal institutions while fostering alienation. More generally, civil and criminal legal systems that penalize attachment to core social institutions are likely to heighten legal anomie by encouraging system avoidance. We expect these effects to be most pronounced in race-class subjugated communities with exclusionary policy design features. To the extent that such compounding cyclical processes between legal anomie and system avoidance operate in other state institutions with alienating policy designs, we believe this model will be transferable to other research contexts in sociolegal studies.

In line with previous scholarship, we view these processes as reproducing social inequality and as “a feature of locations that are marked by state disinvestment and disempowerment” (Sendroiu et al. 2022). Following Bell’s legal estrangement model, we view structural exclusion as key to understanding individual perceptions of and behavior towards the law and legal actors. In this way, we conceptualize the entanglement of child support and criminal debt as a structure that, in part, shapes the experiences, attitudes, and behaviors of those enmeshed within this institutional nexus. This is consistent with classic formulations of legal cynicism and the role of social exclusion in the formation of attitudes. As Sampson and Bartusch (1998:801) noted, “[p]erhaps we should not be surprised that those most exposed to the numbing reality of pervasive segregation and economic subjugation become cynical about human nature and legal systems of justice.” These findings also echo recent scholarship that illustrates how policy design can create a series of burdens on policy subjects that routinely exacerbate social inequalities (Herd and Moynihan 2018; Herd et al. 2023; Parolin et al. 2023; Paik 2021; Ray et al. 2023).

While this study uses rich and unique qualitative data, some important limitations should be noted. First, our study is limited to one site: Minnesota. We selected Minnesota because, in comparison to many other U.S. sites, it is somewhat less punitive in how it handles both child support and monetary sanction debts. However, Minnesota is also unique in other ways such as having a stronger social-safety net than many states and a whiter population than many other states. Second, our sample is largely male and primarily white, and while this is reasonably consistent with the demographics of the population of persons with dual debt in Minnesota, it does not allow for us to examine how these experiences may be distinct for women and/or people of color. Third, we recruited our participants using a purposive sampling strategy (through online postings and connections with community partners), so the perspectives of persons with dual debt who were not connected to social media or community reentry organizations were not captured.

For future research, we encourage scholars studying the dual debt phenomenon to consider Grigoropoulou and Small’s (2022) recommendation to use qualitative data to better understand large-scale administrative data on human behavior. Such efforts help researchers evaluate administrative data quality and ensure that interpretations reflect the experiences of those impacted by multiple, overlapping systems of surveillance, including the racialized, gendered, and localized nature of dual debt experiences. Incorporating this form of “small data analysis” is especially crucial for understanding the relationship between experiences of legal anomie and system avoidance, in which administrative data sources may only provide intermittent or fragmented coverage (Bjerre-Nielsen and Lind Glavind 2022; Grigoropoulou and Small 2022).

Our findings contribute to greater knowledge about how alienating policy designs exacerbate both legal anomie and system avoidance through mutually reinforcing processes of distrust and disengagement from social institutions. More specifically, this study advances scholarship on the strategies and perspectives of people indebted to both punishment and welfare institutions. We add to the growing literature on the collateral consequences of criminal legal involvement, specifically the impacts of criminal legal debt (Harris 2016; Horowitz et al. 2022; Slavinski and Pettit 2021). More broadly, this research draws from and contributes to scholarship on poverty governance, specifically how the penal and welfare systems act as interconnected forms of social control for marginalized populations (Soss et al. 2011; Wacquant 2009). In doing so, we address how the “right” and “left” hands of the state operate in tandem for those under the surveillance of both (Bourdieu 2004).

## Supplementary Material

Appendix B

Appendix A

## Footnotes

1. The theory of legal cynicism has been widely applied to studies of policing (Bell 2016; Campeau et al. 2021; Carr et al. 2007; D. S. Kirk and Matsuda 2011; Phelps et al. 2021), as well as prison reentry (D. S. Kirk 2016), immigrant detention (Gleeson 2021; Ryo 2016), decreased political participation (Lerman and Weaver 2014), and international conflicts (Boutros 2018; Hagan et al. 2016).

2. Bell’s reconceptualization of legal cynicism as a form of estrangement caused by structural dispossession has influenced scholarship in a variety of legal contexts, including policing (Huebner and Giuffre 2022; Nguyen and Roman 2023; Powell and Phelps 2021), immigration (Ramirez 2021; Van Natta 2023), prisoner reentry (Halushka 2020), political marginalization and disenfranchisement (Remster and Kramer 2023), and monetary sanctions (Spencer-Suarez and Martin 2021).

3. Although we conducted our recruitment and interviews in the state of Minnesota, we did not limit our sample to those whose child support debts were from Minnesota. Several of our interviewees had child support debt from other states including Tennessee, Florida, and Wisconsin.

4. The maximum percent of a person’s disposable income that can be garnished for child support varies depending on whether the individual has a spouse or dependent children and the age of the debt. For an individual who is not supporting a spouse or any dependent children and debt over 12 weeks old, the maximum is 65%. If the debt is less than 12 weeks old, the maximum garnishment is 60%.

5. Interviews were conducted with approval from the University of Minnesota Institutional Review Board (ID 1604S86204). In order to protect the confidentiality of our participants we use pseudonyms and change any other identifiers in the results presented below.

6. Specifically, we consider “sexist or gendered critiques” unique, as well as critiques of systems other than monetary sanctions or child support. Both of these subcodes will be explored in greater depth in future studies.

7. Although nonpayment can directly lead to incarceration in many states, Minnesota formally prohibited incarceration due to nonpayment of CLFOs in 2009 (Harris et al. 2017; Sec. 609.104 MN Statutes 2023). Civil contempt for unpaid child support may result in jail time – nine participants reported civil contempt or criminal nonsupport charges, three of whom stated that they had actually been jailed.

8. For example, some courts are implementing new forms of communication to improve high failure to appear rates. Fishbane et al. (2020) estimate that 40 percent of defendants did not attend their court hearing in New York City in 2015 and find that more effective court communications (such as text reminders) improve these rates. However, Kohler-Hausmann (2020) argues this study misrepresents the work of misdemeanor courts, where administrative burden and procedural hassle are core to its penal function (see also Kohler-Haussmann 2019). Thus, people may avoid these institutions not only because of lack of information but as a strategy of limited resources.
