# Supplementary material for: “It’s two separate systems that … keep you under a thumb”: dual debt in the child support and criminal legal systems
Source: Law Soc Rev. Author manuscript; Available in PMC 2025 Dec 1. (PMC11887652; doi:10.1017/lsr.2024.47)
Supplement: Appendix B [file NIHMS2050981-supplement-Appendix_B.pdf]

Appendix B. Dual Debt Coding Scheme

Name	Description	Files	References
Fairness	Attitudes or Perceptions about Fairness	21	48
CS - Child Support Fairness	Respondent expresses thoughts, attitudes, and/or feelings about the fairness of any aspect of child support (e.g. perceptions of bias, discrimination, issues of transparency and accountability)	30	378
LFO - Legal Financial Obligation Fairness	Respondent expresses thoughts, attitudes, and/or feelings about the fairness of any aspect of monetary sanctions (e.g. perceptions of bias, discrimination, issues of transparency and accountability)	29	158
Future Plans, Expectations, and Goals	Respondent shares aspirations, plans, expectations, goals, concerns, etc. about their future, particularly as it relates to debt (likely to be double-coded with IMPACT OF DEBT). This may include but is not limited to plans for seeking/obtaining employment, job training and certification, achieving financial stability, rebuilding personal relationships; may also refer to barriers to achieving goals.	28	124
Institutional Cynicism and criticism	Refers to instances in which respondents share skeptical, critical, and otherwise negative views of institutional systems and actors, particularly child support and criminal-legal authorities (but this may also refer to other systems, such as public assistance/social welfare)	30	669
Ambiguity in Law		18	35
Amounts too great		23	96
Counterproductive		26	119
Critique of different system	critique of system other than monetary sanctions or child support	21	83

Name	Description	Files	References
Critiques about specific fees or costs		19	56
Delay or slow-moving system		7	18
Difficulty navigating systems or confusion		27	128
Interest or other poverty penalties		13	25
Never-ending or Overpowering		23	121
Public defender	Any critiques of public defender	17	29
Resistance		15	29
Sexist or gendered critique		25	105
Skepticism		16	39
State profit		20	52
Structural Critique		14	39
Off the Books Income (non-familial)	Any discussion of respondent's income not obtained through formal employment, other formal channels (e.g. public benefits), or family/friend contributions. Also refers to discussion of past, current, or future work outside the formal Labor market; this may include illicit income.	25	89

Name	Description	Files	References
Personal Networks Conflict or Strain	Conflict or issues with any family members or loved ones generally, and with custodial parents and/or children specifically. “Issues” may include estrangement, custody disputes, etc.	30	316
System Avoidance	Respondent talks about avoiding any formal, record-keeping systems and institutions. These systems/institutions may be public or private and include (but are not limited to) on-the-books employment, criminal courts, civil courts, law enforcement, public bureaucracies/agencies (e.g. public assistance, DMV), financial institutions, schools, health and mental health care providers, etc.	29	143
