# Supplementary material for: “It’s two separate systems that … keep you under a thumb”: dual debt in the child support and criminal legal systems
Source: Law Soc Rev. Author manuscript; Available in PMC 2025 Dec 1. (PMC11887652; doi:10.1017/lsr.2024.47)
Supplement: Appendix A [file NIHMS2050981-supplement-Appendix_A.pdf]

Appendix A. Dual Debt Interview Guide

Introduction

Thank you for taking the time to meet and for agreeing to be interviewed. We appreciate your helping us with this study.

Before we get started, I should introduce myself. I'm… [name, title/university affiliation, research interests, etc.].

Over the last few years, the other researchers on this project and I have been working together, studying criminal justice fines and fees. As we interviewed people about their experiences with court debts, we became aware of the fact that lots of people with court debt also have child support debt. So that's what this project is about. We realize that having these debts can sometimes make people's lives very hard, and we want to learn more about it. We want to understand what it's like having both criminal justice debt ***and*** *child support debt—what people's thoughts and opinions about these debts are, and how they deal with them. We are really interested in your story.*

If it's ok with you, I'm going to audio record our conversation. This is so that I can really focus on what you're saying instead of writing notes. It will also help me stay faithful to what you've said when I go back and review the interview. Your name and any information that may be used to identify you will be kept private [more information on confidentiality in informed consent form]. *Is audio recording ok?*

General Background

1. To get things started, why don't you tell me a bit about yourself?

   How long have you lived in [city]? In Minnesota? Where did you grow up?

   Where did you go to school? How far did you go in school?

   Can you tell me about your family growing up—who raised you, whether you had brothers and sisters…?

Housing and Living Situation

2. Let's talk about where you're living now.

   What's your current housing situation—apartment, house, transitional/treatment facility, shelter, etc.?

   Do you pay monthly rent (or mortgage)? If so, how much?

   Who do you live with (partner/spouse, family, friends, etc.)?

   Have you ever had any problems with housing (evictions, homelessness, discrimination, etc.)?

Employment, Income, and Benefits

3. Before we get into talking about your court and child support debts, it will be helpful for me to understand your income and work situation.

   Are you working right now?

      If yes, probe type of employer and employment, job duration, on or off the books, wage or salary income, full or part time, regular or irregular hours

      If no, probe how long participant has been without work, most recent work experience, issues/barriers related to finding or keeping employment

   Do you have any sources of income other than regular work—odd jobs, illicit income, public assistance (MFIP, GA, MSA), SSI (Supplemental Security Income), SSDI (Social Security Disability Insurance), etc.?

   If you earn income off the books, would you describe this more of a choice or a necessity?

Do you have health coverage—employment benefits, Medicaid/Minnesota Care, Medicare, etc.?

Do you receive financial or other material help from family or friends?

How much money do you need in order to get by in a given month?

Aside from criminal justice and child support debt, do you have any other financial debts—medical, student, credit card, etc.?

Children and Family

4. Are you single, married, divorced or separated (currently)?

    (If not married) Have you ever been married?

    Are you in a relationship? If yes, how long have you been with your partner?

5. Tell me about your children.

    How many kids do you have?

    What are their ages, genders, and grade levels in school?

    Where and with whom do your kids live?

    What are your relationships with your kids like?

    How would you say they're doing, in terms of their wellbeing?

    [Specific probes: ***school***, friends, health and mental health]

6. How many partners/co-parents do you have children with?

    What is/are your relationship(s) with your kids' custodial parent(s) like?

Child Support Obligations

7. Tell me about your experience with the child support system. [and your child support obligations] .

    How many child support orders do you have, for how many kids?

    How much do you owe on a monthly basis? How much do you usually pay?

    When and how were your child support orders imposed?

    Did you go to family court? What was the process/experience like?

    How was the order amount determined?

    Do you know whether the order was established because the other parent receives public assistance? (Probe their knowledge of the difference between child support in public assistance and non-public assistance cases.)

    When your order was set, did someone explain the order, your obligations, and how it all works to you?

    If yes, how was that information communicated to you?

    Did you feel then like you had an adequate understanding of the order and all the procedures involved?

    Right now do you feel like you have a clear understanding of how child support orders are set and enforced?

    Are there any aspects of child support that are confusing or that you'd like to understand better?

8. Do you provide support to your children in other ways, beyond your legal child support order, such as other financial support, in-kind (non-financial, e.g. clothes, school supplies, food) support?

Do you prefer providing one kind of support to another?

Do you know if your kids' custodial parent has any preferences about the type of support you provide?

9. Let's go back to talking about your formal child support order. What is your procedure for making child support payments?

   Do you know how much you owe?

      If yes, how do you find out what you owe? If no, do you know how you would find out how much you owe?

      Do you receive regular payment notices?

   Do you have payments automatically deducted from your work, benefits, or any other type of income?

   Does child support enforcement contact you? Can you describe your interactions with child support enforcement?

10. How much do you owe in child support arrears?

    How did the debt accumulate? (Probe time it took to accumulate and factors contributing to debt build-up.)

       Were you aware that you were accumulating arrears?

    Did child support enforcement contact you about your arrears? What happened?

11. Have you ever tried to get a child support order modified?

       If yes, what happened?

       If no, why not?

    What do you know about child support modification and how it works?

12. To the best of your knowledge, what are the possible consequences for not paying child support?

    Have any of the following happened ***because of your child support debt***? (Check those that apply to you.)

____ I have been to court

____ I have faced civil contempt-of-court charges for nonpayment

____ I have had criminal charges for nonpayment

____ I have received a parole/probation violation

____ I have been re-incarcerated

____ I have had my driver's license suspended

____ I have had a lien placed on my assets

____ I have had contact with the IRS

____ I have had one or more account frozen

____ My credit score has been affected

____ I have had difficulty getting credit cards, loans, etc.

13. Have you ever gotten legal counsel or assistance in your child support case(s)?

Criminal Justice Involvement and Debt

14. Let's shift gears and talk about your criminal justice experiences and debt. To start, would you mind explaining a bit about past convictions?

Can you talk about your misdemeanor convictions? Felony convictions? (Probe number and years of convictions, offense categories)

Were you incarcerated in jail or prison for any convictions?

15. What court costs, restitution, and other financial penalties were imposed on you as a result of your criminal convictions?

    How much do you currently owe, for which financial penalties?

    When the financial penalties were imposed, who told you about what you owed and what it was for?

    Did you feel like you understood the financial penalties or was it confusing?

16. If you are under criminal justice supervision right now, can you explain what kind (e.g. probation, parole, halfway house) and the conditions involved?

    Do you have to pay supervision fees? How much? Do you have to pay for any classes or treatment

17. What is the procedure for making payments on your court debts?

    How do you find out what you owe?

    Do you receive regular payment notices?

    Do you have payments automatically deducted from your work, benefits, or any other type of income?

    Does the court or anyone else contact you about what you owe?

18. To the best of your knowledge, what are the possible consequences for not paying criminal justice debt?

    Have any of the following happened ***because of your criminal justice debt***? (Check those that apply to you.)

____ I have been to court

____ I have received a parole/probation violation

____ I have been re-incarcerated

____ I have had my driver's license suspended

____ I have had a lien placed on my assets

____ I have had contact with the IRS

____ I have had one or more account frozen

____ My credit score has been affected

____ I have had difficulty getting credit cards, loans, etc.

19. What kind of legal counsel did you have in your criminal case(s)?

    What was your criminal court experience like?

Experiences and Perspectives

20. How do your debts affect your day-to-day life?

    How do your debts affect decisions you make about work, money, or other aspects of your life? Your expectations or plans for the future?

    How might your life be different if you didn't have these debts?

21. Compare the impact of child support debt on your life with the impact of criminal justice debt. Do they affect you in different ways or play different roles in your life?

How do you manage your child support debt and your criminal justice financial obligations? Describe a practical strategy that you use to either deal with or put off the debts. Why do you use this strategy?

22. What are your thoughts about the fairness of child support debt?
    - Do you think differently about the fairness based on whether the child support case was initiated because the custodial parent receives public assistance?
    - What do you think about the fairness of child support collection procedures?
    - What do you think about the fairness of child support nonpayment sanctions?

23. What are your thoughts about the fairness of criminal justice financial penalties?
    - Do you think differently about the fairness of different types of court debt?
    - What do you think about the fairness of collection procedures?
    - What do you think about the fairness of nonpayment sanctions?

24. We're trying to understand how criminal justice debts might affect other family members as well. Do you think these debts have affected any of your family members?
    - Your children? [*Probe*: effects in school, friends, mental health, etc.]
    - The custodial parent/guardian? [*Probe*: financial or other strain]
    - Parents (of the debtor) [*Probe*: financial or other strain]
    - Others (e.g. family, friends, current romantic partner)
    - How has this impacted your relationship with your [family member]?
    - [*Probe*: tough decisions, trade-offs, etc.]

25. How would you describe your experience with the child support system?

26. If you could make changes to the child support system, what would they be?

27. How would you describe your experience with the criminal justice system, as it relates to fines, fees, and other financial sanctions?

28. If you could make changes to how the criminal justice system handles financial penalties, what would they be?

29. Is there anything else you'd like to share about your debt experiences? Or anything we haven't asked that you think is important?

Demographics

30. To wrap up, I have just a few more quick questions about you.
    - How old are you?
    - What is your gender?
    - How would you describe your race/ethnicity?

Wrap-up

Those are all of the questions that I have for you. Do you have any questions for me?

Thank you for taking the time to meet and for sharing your thoughts and experiences.
